# Efficacy of fractional laser, radiofrequency and IPL rejuvenation of periorbital region

**DOI:** 10.1007/s10103-021-03329-7

**Published:** 2021-05-14

**Authors:** Anna Kołodziejczak, Helena Rotsztejn

**Affiliations:** Department of Cosmetology and Aesthetic Dermatology, Faculty of Pharmacy, Medical University of Łódź, Muszyńskiego 1 Street, 91-151 Łódź, Poland

**Keywords:** Fractional laser, Radiofrequency, Intense pulsed light, Skin rejuvenation, Periorbital area, Cutometer

## Abstract

The purpose of this study was to assess skin elasticity, the reduction in the number and the depth of wrinkles and changes in the other skin defects (bags under the eyes, dark circles under the eyes, skin hyperpigmentation in the ageing eye area following the use of non-ablative fractional laser, bipolar radiofrequency and intense pulsed light). Moreover, the study was also comparison which device brought better results than the others. This study included 71 patients (66 women, 5 men), aged 33–63 years (the average age was 45.81 years) with skin phototypes II and III. Twenty-four patients received five treatment sessions with a 1410-nm non-ablative fractional laser in 2-week intervals, 23 patients received five treatment sessions with a bipolar radiofrequency in 1-week intervals and 24 patients received five treatment sessions with an intense pulsed light in 2-week intervals. The treatment was applied on the skin in the eye area. The Cutometer (Courage + Khazaka electronic) reference test was used as an objective method for the assessment of skin elasticity. A questionnaire was used to compare baseline state with changes that occurred after the series of treatment sessions. The results of cutometric measurements of R2, R6 and R7 parameters and the results of questionnaires indicated that non-ablative fractional laser therapy, bipolar radiofrequency and intense pulsed light improved skin elasticity. Of the three treatments, the most significant percentage improvement in the R6 parameter was demonstrated by non-ablative fractional laser therapy which gave better final results than the other methods (*p* < 0.0001). No other statistically significant relationships were found between RF and IPL. In the (subjective) opinion of study participants (questionnaire), all used methods resulted in the reduction of the amount and the depth of wrinkles. However, they did not observe significant impact of individual treatment method on the signs of skin ageing, including discolorations within eye area, bags (fatty hernia), dark circles (vascular/pigmentary) and oedema (predisposition to water retention). Non-ablative fractional laser therapy, bipolar radiofrequency and intense pulsed light improved skin elasticity and the reduction of wrinkles. The most significant improvement of elasticity was demonstrated by laser therapy. It seems necessary to expand the group with the effect of individual treatments against bags and dark circles under the eyes.

## Introduction

For decades, scientists have been interested in skin ageing, perhaps because it is a relatively easily available research model. Such objective assessment is an important source of knowledge about the methods, results and possible side effects of anti-ageing treatments. Specific skin structure (slight thickness, tenderness, variation and subcutaneous tissue displacement, remodelling of the orbital bone, superficial arrangement of blood vessels), as well as aggravation by age-related symptoms, makes the eye area one of the most difficult places to perform aesthetic procedures [1].

Non-ablative fractional laser therapy is a procedure in which fragmental thermal damage is induced within the exposed skin. Radiofrequency treatment, i.e. the use of radiofrequency electromagnetic waves, causes local hyperthermia by converting electrical energy into intracellular heat following absorption by individual skin structures; the wavelength and light dose are adjusted to enable the photorejuvenation of ageing skin and to reduce pigmentation and erythema changes. The treatment induces a thermal response in the dermis, thus promoting connective tissue remodelling and the formation of new collagen, resulting in improved skin elasticity.

The purpose of this study was to assess the efficacy of the use of non-ablative fractional laser, non-ablative radiofrequency (RF) and intense pulsed light (IPL) in the treatment of signs of ageing within eye area (skin elasticity and viscoelasticity). The study was also aimed at finding the most effective of the compared devices. Moreover, we also evaluated the reduction in the number and the depth of wrinkles and changes related to other skin defects (bags under the eyes, dark circles under the eyes, skin discolorations) on the basis of questionnaire filled by patient participants.

## Materials and methods

### Material

This study included 71 patients (66 women, 5 men). The mean age of the group was 45.81 years (range: 33–63), and most participants demonstrated skin phototype II or III and I, II and III degree of wrinkling according to Fitzpatrick. Patients showed various comorbid ageing symptoms, the reduction of which was also assessed in this study (Table 1).

**Table 1:** The characteristics of the study group, judged by clinicians

Treatment	Mean age	Number of patients	“Bags” under the eyes	Transient oedema	Falling eyelids	Discolorations	“Shadows” under the eyes
Laser	44.6	24	3	10	4	7	9
RF	44.8	23	6	6	8	5	10
IPL	48.4	24	3	5	2	10	8

We informed potential participants about this research via the distribution of recruitment materials. Also we recruited participants in the cosmetology and aesthetic dermatology department. All subjects provided written informed consent to take part in the study. Following initial enrolment, all participants took part in an interview qualifying for the procedures used in this study. A total number of 8 people were excluded after the qualification procedure: 2 participants (the lack of assessed defects), 4 (medical contraindications: labial herpes, pregnancy 2 × , keratitis), an aesthetic procedure in the course of research (dermal fillers for crow’s feet), 1 (advanced age). Recruitment was carried out in several stages. The type of treatment was assigned randomly. The subjects were assigned to the method depending on the time of reporting. The group subjected to laser therapy was implemented first, the second group was RF and the third group was IPL. The selection was independent of clinical, demographic and other (apart from assumed) characteristics. The series of individual treatments lasted 6 months. All participants completed the study. Study participants received information leaflets on postoperative management in order to reduce the risk of possible occurrence of side effects. None underwent any other procedures based on cosmetology or aesthetic dermatology.

### Methods

The first group of participants were treated with a 1410-nm non-ablative fractional diode laser (Emerge, Palomar). The participants were subjected to five non-ablative fractional laser treatments at 2-week intervals. Coagulation columns did not exceed 450 microns. The laser causes coagulation of 2.5% of the scanned skin surface. It is classified as a Class II Medical Device (USA), Class Ibis (UE), Class 1 M compliant with IEC 60,825–1:2007. Patients wore external overlay eye shields for eye protection during the treatment. The parameters were selected individually based on Fitzpatrick phototype (Table 2), with the aim of decreasing existing hyperpigmentation and reducing the risk of post-treatment discoloration. Hence, lower energy density (mJ/lC), concentration pattern and numbers of micro-columns in the scanning surface were used in patients with a darker phototype and with visible areas of hyperpigmentation.

**Table 2:** Parameters used during non-ablative fractional laser treatment

Surgical parameters	Energy mJ/μC	Pattern (density) scan grid	No. of micro-columns within scanned area	Pitch (mm)	Recommendations based on Fitzpatrick skin phototype	Number of patients
A	20	8 × 5	40	1.5	Medium and darker III phototype	7
B	25	9 × 6	54	1.3	I–II phototype and brighter III	9
C	30	10 × 7	70	1.1	I–II phototype	8

The second group of participants underwent non-ablative bipolar radiofrequency (RF) (Thermalipo II, Thermamedic) with an AMPLI (Automatic Multi-Frequency and Low Impedance) device. The most commonly used frequencies in the present study were 0.9 MHz 0.6 MHz, 1.8 MHz 1.2 MHz, 2.7 MHz 1.8 MHz, 3.6 MHz and 2.4 MHz. Bipolar facial applicator distributes the energy of radio waves to a tissue depth of 4–6 mm. During this procedure, the temperature of the epidermis can reach 40–42 °C. During the procedure, a conductive gel was used. At the beginning of the treatment, high skin resistance is detected by electrodes and a low frequency is used. RF energy affects the deeper layers of tissue until erythema appears. As the resistance decreases, the device starts to emit higher frequencies that heat up the upper layers of the skin. When the nociceptors feel warmth, the treatment is terminated. Patients underwent five bipolar radiofrequency treatments at weekly intervals, this being the most widely recommended interval between treatments.

In the third group, IPL (intense pulsed light) treatments (MIMARI HM-IPL-B1) were performed using a 530-nm cutoff filter: a beam with a wavelength of 530–1200 nm was emitted. The energy density (fluence) was 10 to 50 J/cm^2^, pulse sequence: 1, 2, 3, 4, 5; time interval between pulses: 5–60 ms; pulse duration: 2–15 ms; the frequency of repetitions: 0.3–1 Hz. Light spot size was 15 × 35 mm. Cold water, air and semiconductor cooling were used as cooling system. Cooling temperature was − 4 to 0 °C. The treatment area was protected with a conductive gel for laser therapy. Patients wore external overlay eye shields for eye protection during the treatment. Five IPL sessions were performed at 2-week intervals. The treatment parameters were adjusted according to the phototype and response to treatment (Table 3).

**Table 3:** Parameters used during intense pulsed light treatment

Treatment parameters	Energy J/cm^2^	Sequence of impulses	Impulse delay	Impulse duration	Adjustment to skin phototype	Number of patients
A	16–20	3–4	18–30 ms	4–6 ms	Medium and dark III according to Fitzpatrick	5
B	20–26	2–3	15–18 ms	4–8 ms	Phototype II and light III according to Fitzpatrick	15
C	26–30	2	10–15 ms	7–10 ms	Phototype II according to Fitzpatrick	4

An MPA 580 Cutometer (Courage + Khazaka electronic) was used to assess the mechanical properties of the skin before and after the procedure using the following parameters: measurement mode 1, negative pressure 450 mbar, on-time 3 s, off-time 3 s, repetitions 3. In order to limit the impact of external factors (including cosmetics), the subjects did not use any cosmetics for 6 h before the treatment and their skin was cleaned with warm water and a sterile swab about 5 min before the procedure: skin hydration has an important influence on the R6 parameter, which indicates the relative contribution of viscoelastic, viscous and elastic deformation to total skin deformation. During the measurement, the skin is sucked into a probe hole and mechanically deformed by a fixed, specific vacuum. The depth of skin sucked into the probe is measured by an optical system built into the probe. R2 and R7 parameters were used to assess the effectiveness of the treatment: R2 is a measure of total skin elasticity, including viscosity deformation, and is the most commonly used approach to determining skin ageing, while R7 is an indicator of immediate contraction following a complete deformation, and is related to skin flexibility: values closer to 1 (100%) indicate greater skin flexibility.

Cutometric measurements were performed immediately before the first treatment and then 2 weeks after the fifth (final) treatment.

**Fig. 1:**
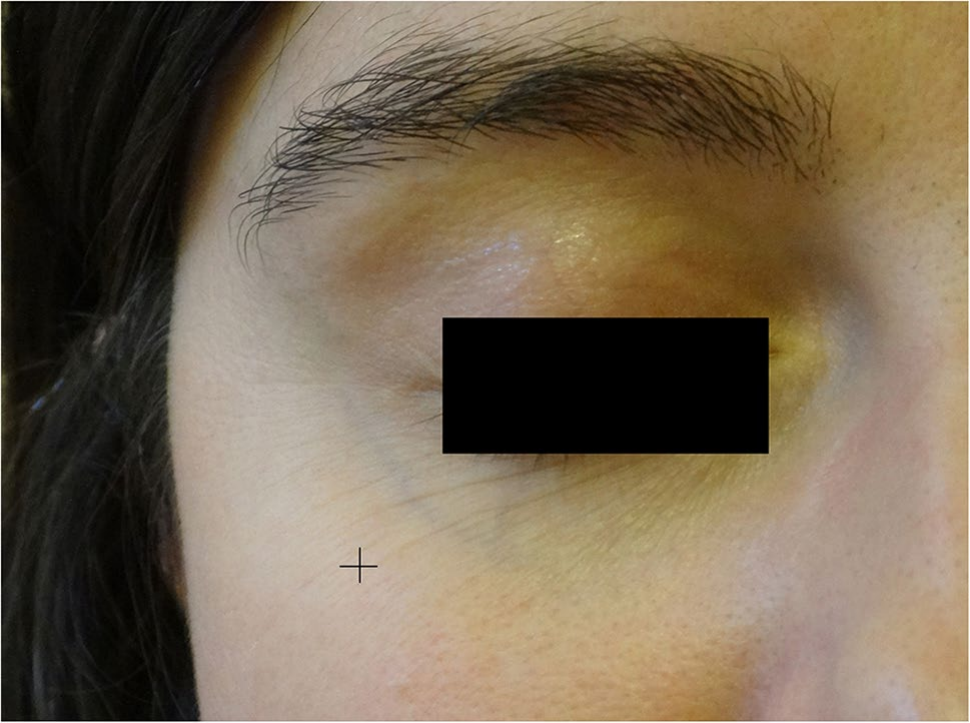
Site of measurements

Measurements were made at a point located 2 cm below the outer corner of the eye (Fig. 1).

In addition, all study participants were surveyed 2 weeks after the final procedure to evaluate their opinion of the effectiveness of treatment. In addition, supplementary questions were asked during the final cutometric measurement, i.e. 3 months after treatment, to account for the time taken for skin remodelling (collagen) to take place and to rule out the influence of various factors on skin pigmentation. Participants were asked to assess their degree of satisfaction with the course of treatments and effectiveness of the procedure. The questionnaires used in the study were not anonymous since their results had to be correlated with the results of measurements.

### Statistical analysis

The MPA (Multi Probe Adapter/Cutometer) was connected to a compatible computer for testing. The measured values can be displayed as bars, curves or numerical data. Here, numerical data were used. All data were stored in one database and then transferred to a spreadsheet (Microsoft Excel®) to perform statistical analysis.

Descriptive findings are presented as simple proportions [%] or means and standard deviations (mean ± SD). The normality of the data distributions was verified with the Shapiro–Wilk test, and the homogeneity of variance was tested with Levene’s test.

In Table 4, comparison of the change in measured parameters for (peak) measured as % for the measurement before and after the series of treatments was designated on the basis of: $((\mathrm{After}-\mathrm{Before})/\mathrm{Before})*100=\mathrm{\%}$. To estimate the significance of differences between selected groups (laser, RF, IPL), one-way ANOVA was used with Tukey’s post hoc test.

**Table 4:** Comparison of the change in measured parameters for (peak) measured as % for the measurement before and after the series of treatments. It was designated on the basis of: ((After-Before)/Before)*100=%

	Method I (laser)*		Method II (RF)^#^		Method III (IPL)		Significance
R2 (before)	0.351 ± 0.125		0.380 ± 0.096		0.323 ± 0.096		–-
R2 (after)	0.504 ± 0.165		0.507 ± 0.096		0.500 ± 0.084		–-
R2 (%)	57.4 ± 71.8		38.3 ± 32.2		69.3 ± 58.6		*P* = 0.180
R6 (before)	0.681 ± 0.346		0.353 ± 0.124		0.561 ± 0.190		–-
R6 (after)	0.322 ± 0.143		0.336 ± 0.125		0.634 ± 0.180		–-
R6 (%)	− 48.4 ± 19.9		*** − 2.4 ± 22.9		***23.0 ± 49.1^#^		*P* < 0.0001
R7 (before)	0.176 ± 0.118		0.134 ± 0.061		0.148 ± 0.057		–-
R7 (after)	0.227 ± 0.116		0.211 ± 0.045		0.252 ± 0.050		–-
R7 (%)	63.4 ± 90.5		87.6 ± 88.9		102.1 ± 101.4		*P* = 0.359
p for R6		Method I (laser)*		Method II (RF)^#^		Method III (IPL)	
Laser		–-		*P* < 0.0001		*P* < 0.0001	
RF		*P* < 0.0001		–-		*P* = 0.031	
IPL		*P* < 0.0001		*P* = 0.031		–-	

Results were presented as mean ± SD. One-way ANOVA followed by Tukey’s post hoc tests was used to determine statistical significance

*P < 0.05; ***P < 0.001 vs method I (laser)

^#^*P* < 0.05 vs. method II (RF)

In Table 5, a statistical analysis was used to indicate how many people reached the thresholds of specific % effectiveness (10%, 25%, 50%, 75%, 100%, 200%). It was calculated what % of people achieved a certain increase from the initial value. Qualitative data was analysed with the chi-square test or Fisher’s exact test where needed (Table 5).

**Table 5:** Subjective percentage assessment of wrinkle reduction by study participants

	0–20%	21–50%	51–70%	71–100%
Non-ablative fractional laser	8	10	6	0
Bipolar radiofrequency	9	10	4	0
Intense pulse light	8	14	2	0

The research project and its procedures were approved by the Bioethics Committee of the Medical University of Lodz (Protocol No. RNN/152/14/KE).

## Results

Percentage change in R2, R6 and R7 values between the two time points was calculated: the first being before the series of treatments, and the second after five sessions of treatment, i.e. 2 weeks after the final treatment (Table 4). Of the three treatments, the most significant percentage improvement in the R6 parameter was demonstrated by non-ablative fractional laser therapy, which gave better final results than the other methods (*p* < 0.0001). No other statistically significant relationships were found between RF and IPL.

A statistical analysis was used to indicate the thresholds of specific % effectiveness. It was calculated what % of people achieved a certain increase from the initial value. Percentage change in R2 and R7 values before the treatment vs. after a series of five treatments was not statistically significant. However, for the R6 parameter, a statistically significant (*P* < 0.0001) 50% improvement in relation to the baseline value was achieved in 11 patients (45.8% of patients) who underwent non-ablative fractional laser therapy.

Similar percentage changes in wrinkle reduction were noted for all three methods by the participants in the questionnaire. The details of this assessment are presented in Table 5. The survey results were not analysed statistically.

The questionnaire concerned the degree of wrinkle number reduction and their depth, as well as the improvement of skin elasticity. Twenty-one out of 24 patients subjected to non-ablative fractional laser treatment observed significant improvement of elasticity of the skin around eyes and eyelids. In the group undergoing bipolar radiofrequency, a considerable proportion of participants “had no opinion” on the effects of therapy. A large portion of participants observed improvement of skin elasticity, and reduced number and depth of wrinkles. In the IPL photorejuvenation group, the majority of the subjects reported the improvement of elasticity, however, only few of them observed a reduction in the amount and depth of wrinkles (Table 6).

**Table 6:** The assessment of treatment efficacy by participants

	Definitely yes	Rather yes	I have no opinion	Rather no	Definitely no
Non-ablative fractional laser					
Reduction in the amount of wrinkles	6	10	6	2	0
Reduction in the depth of wrinkles	8	9	5	2	0
Improvement in skin elasticity and tension	8	13	3	0	0
Bipolar radiofrequency					
Reduction in the amount of wrinkles	6	7	8	2	0
Reduction in the depth of wrinkles	4	9	9	1	0
Improvement in skin elasticity and tension	4	13	6	0	0
Intense pulse light					
Reduction in the amount of wrinkles	4	10	5	5	0
Reduction in the depth of wrinkles	1	12	8	3	0
Improvement in skin elasticity and tension	10	12	1	1	0

* “Rather yes” indicates that the participant is not 100% certain

**Fig. 2:**
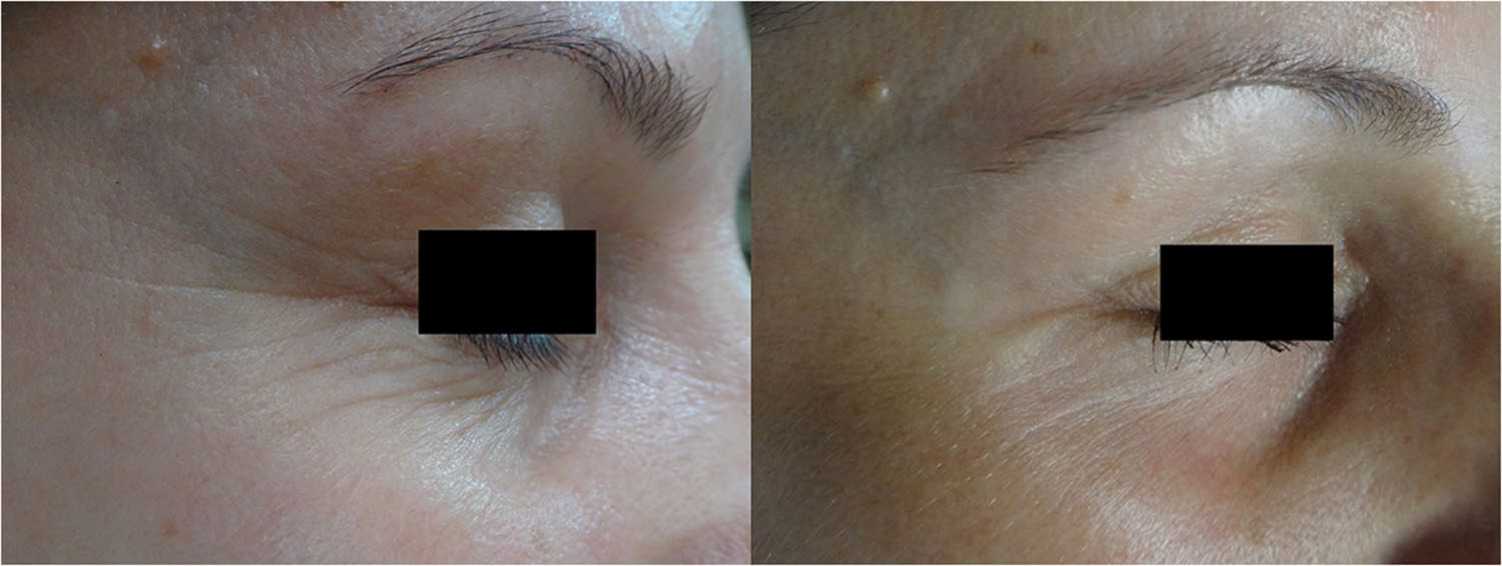
Patient AB, age 42 before the first treatment non-ablative, fractional laser session after the last one

**Fig. 3:**
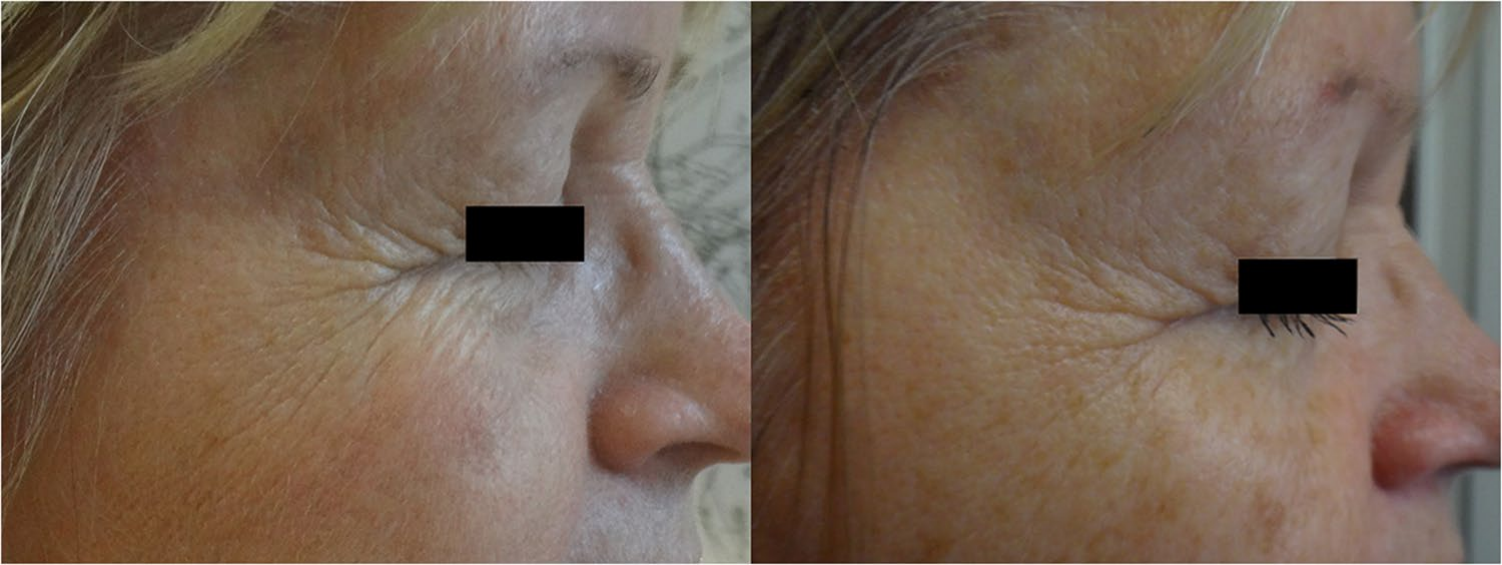
Patient CD, age 58, before the first session of treatment with non-ablative RF and after the last one

**Fig. 4:**
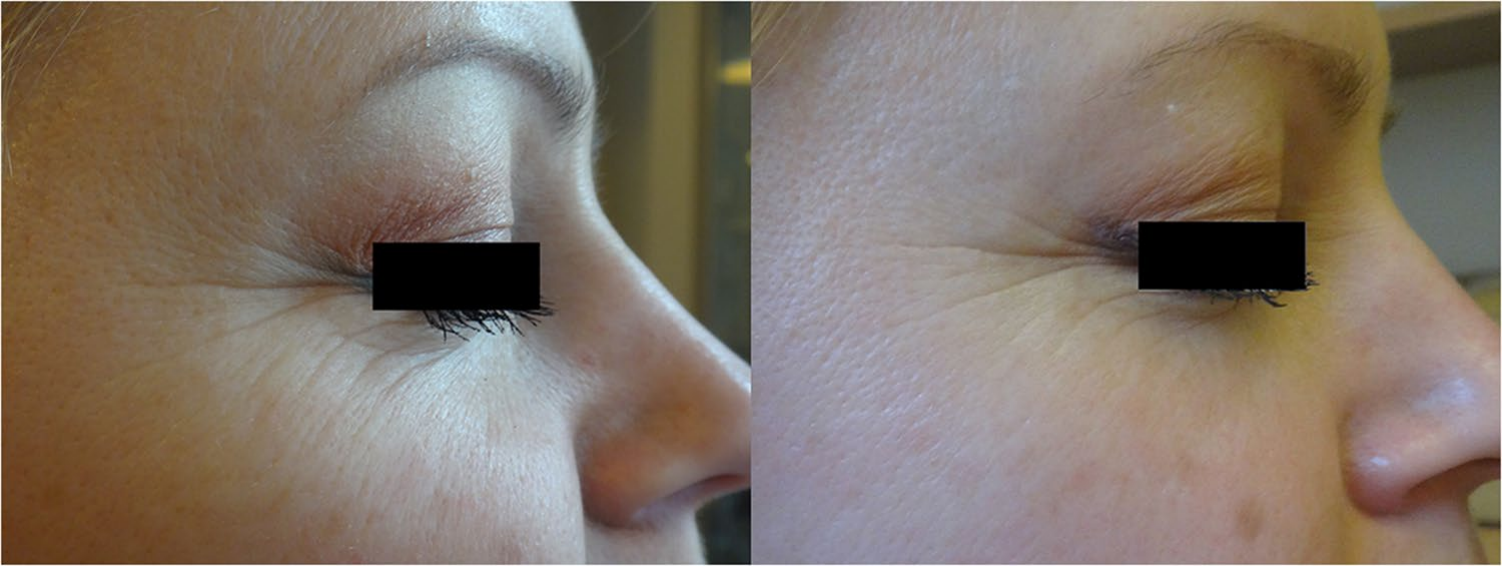
Patient EF, age 43, before the first session of treatment with intense pulsed light session and after the last one

Photo documentation is often used to compare clinical state of skin before and after the series of treatments (Figs. 2, 3 and 4).

Questionnaires also enabled the analysis of the impact of individual treatment techniques on symptoms of skin ageing, such as hyperpigmentantion of eye area, “bags” (fat hernia under the eye), shadows (vascular/pigmentary), oedema (the predisposition to retain water) and significant upper eyelid flaccidity. In the group undergoing non-ablative fractional laser treatment, hyperpigmentantion of eye area concerned 7 persons and 2 of them reported its reduction. The presence of bags under the eyes was reported by 3 participants, 2 of whom observed their reduction. The shadows under the eyes were declared by 9 people, 3 of whom stated their reduction. Ten participants were affected by oedema under the eyes and 5 of them confirmed its reduction. In the group treated with bipolar radiofrequency, the presence of bags under the eyes was reported by 6 participants and 5 of them observed their reduction. The oedema under the eyes concerned 6 people, 1 of whom stated their reduction. This group was not asked about hyperpigmentantion and shadows under the eyes because this device cannot influence this type of change (it is associated with its course of action). In the group treated with intensive pulse light (IPL), hyperpigmentantion within the eye area concerned 10 people and 6 of them reported reduction. Bags under the eyes concerned 3 people, 2 of them stated that bags reduced following treatment. Eight persons claimed to have shadows under the eyes, and none of them observed their reduction. Finally, 5 participants complained to have oedema under the eyes and 2 of them reported its diminishing. This group was not asked about the flaccidity of the upper eyelid, because IPL procedure cannot be performed on the eyelids for safety reasons.

## Discussion

Skin mechanical properties, namely plasticity and elasticity, are guaranteed by the perfect interaction between epidermis, dermis and hypodermis components. The cutometrical measurements seem to be useful to evaluate improvement of elasticity of skin after aesthetic treatments. Numerous studies have confirmed that the process of dermis remodelling takes place in thermally modified tissues. The aim of these systems (non-ablative fractional laser, RF and IPL) was to activate the thermal response in the dermis. It is connected with connective tissue remodelling. The improvement of tension and density of the skin result from contraction of the “old” collagen and stimulation of fibroblasts to produce new fibres (elasticity) and proteoglycans (viscoelasticity). Our present findings indicate that a series of non-ablative fractional laser therapy, bipolar radiofrequency, or intense pulse light treatment significantly affected skin elasticity. These types of treatments are effective methods of rejuvenation of skin around the eyes. These treatments can be used to improve skin elasticity and reduce wrinkles (reduction in the amount and depth of wrinkles) in the eye area. The most significant improvement was demonstrated by non-ablative fractional laser therapy. The greatest effectiveness may be related to the fact that the applied laser therapy effectively affects both the elasticity and viscoelasticity of the skin.

Naouri et al. examined the improvement of skin tension after the application of fractional laser treatment (CO_2_ laser) in a group of 17 women. Cutometric measurement showed significant changes in the following parameters: elasticity (R2 + 5.9%), viscoelasticity (R8 − 9.4%), fatigue (R3 and R9 − 16.2%, − 19.7%, respectively) and thickness R0 − 14.9%) [2]. Kim et al. conducted a Cutometer study to assess the effect of monopolar radiofrequency treatment on skin elasticity; however, their results were statistically insignificant [3]. Shin et al. used R2 and R5 parameters to evaluation of IPL application in a group of 23 women. Also the results of other studies confirm that IPL treatment increases skin elasticity, but does not affect the reduction of wrinkles [4–7].

Our present findings suggest that of the tested therapies, non-ablative fractional laser therapy stimulates the greatest increase in skin viscoelasticity as indicated by Cutometer parameter R6. Kapoor and Saraf demonstrated a relationship between skin hydration and viscoelasticity parameters in a study of the effects of various moisturizing compounds on skin viscoelasticity in 40 subjects. The increase in these values may indicate a decrease in the viscosity of interstitial fluid as a result of increased water content and changes in the composition or structure of proteoglycans [8]. Other authors report that parameter R6 depends on skin hydration [9–11], while others have found that with ageing, changes in skin elasticity coefficient (R2, R7) are more pronounced than those in skin viscoelasticity (R6) [12, 13]. Kruger et al. report a high correlation between R2 and age, and a low one between R6 and age, and recommend that parameters R2 and R7 be used in the assessment of skin ageing [14].

Cutometer evaluation of skin elasticity is a good complement when assessing the reduction of wrinkles and other skin defects. Although some of the cutometric readings were not statistically significant, they nevertheless indicate improved skin elasticity.

The questionnaire filled in by patients is a perfect complement to the clinical assessment. The patients undergoing treatment also completed a survey to subjectively evaluate treatment effectiveness. Young Ji Hwang et al. used an ablative fractional CO_2_ laser to reduce wrinkles and acne scars. Its effectiveness was evaluated on the basis of the percentage reduction in wrinkles observed by the patient, the investigators and independent researchers. Changes were described as follows: 0 degree—no changes, 1 degree—minimal improvement (1–25%), 2 degree—average improvement (26–50%), 3 degree—significant improvement (51–75%), 4 degree—total improvement (over 75%). The effectiveness of the therapy was found to be 2, 3 or 4 degree, respectively [15]. The same scale of patients and investigators assessment was used by Hun Lee et al. in the study concerning the reduction of wrinkles associated with ablative fractional laser treatment [16] and by Tretti et al. in the treatment of stretch marks with non-ablative fractional laser [17]. In the present study, 21 out of 24 persons undergoing non-ablative fractional laser therapy reported improvement in the elasticity of skin around the eyes and eyelids.

Sukal et al. evaluated the influence of a non-ablative fractional laser with a wavelength of 1550 nm on the elasticity of the lower and upper eyelids, on the basis of photo documentation using a 4-point scale. One-third of respondents stated that the efficacy was 75 to 100%, while 50% improvement was reported by 26% of participants. The procedure involved the entire face; it was found that an increase in the elasticity of the adjacent area, i.e. the forehead and cheeks, influenced the appearance of the area around the eyes. Therefore, the authors recommend that improvements in eyelid elasticity should be evaluated only in therapies limited to this area, to exclude the influence of neighbouring areas on eyelid tension. They also note that the procedure was safe in the eyelid area and could be performed without the use of protective glasses [18]. In our study, no long-term side effects were observed in this area following treatment. Bonan et al. report that therapy with CO_2_ fractional lasers significantly reduced upper eyelid flaccidity, widened the eyelids and reduced wrinkles [19].

In the group undergoing bipolar radiofrequency, a significant proportion of people reported having “no opinion” about the effects of therapy. A large portion of participants observed improved elasticity and a reduction in the number and depth of wrinkles. This result could be attributed to the fact that increased skin elasticity reduces the visibility of wrinkles around the eye through the raising of the superciliary arch and upper eyelids [20, 21]. Fitzpatrick et al. report an elevation of the superciliary arch by 0.5 mm in 40 out of 65 people undergoing non-ablative radiofrequency therapy [22].

In the IPL photorejuvenation group, the majority of the participants observed improved skin elasticity, while a few of them reported a reduction in the amount and depth of wrinkles. Hedelund et al. found that IPL treatment improved skin texture, telangiectasia and pigmentation, but had no impact on wrinkle reduction [5]. Photodynamic therapy (PDT) with IPL and photosensitizing compounds, which become toxic to the target tissue when exposed to a specific type of light, can be used to reduce the symptoms of ageing [23].

No significant improvement in the reduction of other skin defects (bags under the eyes, dark circles under the eyes, skin discolorations) has been shown; therefore, in our opinion, these methods are not effective in the reduction of the aforementioned symptoms. However, the information on the main operating principles of these devices implies that they can be used to reduce the above-mentioned defects. Therefore, the results of this study should be confirmed in a larger group.

The perceived impact of the different treatment techniques on skin ageing symptoms, such as eye area discoloration, bags (fat hernia under the eye), shadows (vascular/pigmentary), oedema (prone to water retention),and significant flaccidity of upper eyelid, was reported in the present study. Oedema under the eyes was reported by 10 people, and five of them observed its reduction. Bonan et al. report that laser treatment did not result in the reduction of excessive fat in the lower eyelid and did not diminish muscle hypertrophy around the eyes in a study of 45 subjects. In addition to visible wrinkles and skin flaccidity, some participants displayed characteristic signs of ageing around the eyes, such as a lowering of eyebrow position and fat hernia under the eyes. [19]. Mood et al. used a non-ablative fractional erbium laser with a wavelength of 1550-nm laser to brighten the eye area in one Caucasian woman. The authors demonstrated a reduction in hyperpigmentation and a beneficial effect on other signs of ageing of the eye area, and claim that the use of non-ablative fractional laser therapy to reduce hyperpigmentation was justified [24]. In the case of “shadows under the eyes”, which result from melanin deposition in the upper layers of the dermis, the use of fractional lasers acting at the same depth was shown to produce good effects. Xu et al. found a Q-switched fractional laser to demonstrate good efficacy in the reduction of eye shadows in a group of 30 individuals [25].

In the group treated with bipolar radiofrequency, bags under the eyes were reported by six people, five of whom stated their reduction. The improvement of skin elasticity can have an impact on the reduction of bags and/or oedema related to excessive skin softness [20, 21]. This group was not asked questions about discoloration or shadow under the eyes because radio waves do not affect skin chromophores [26].

In the intensive pulse light (IPL) group, eye discolorations were present in 10 people, and six of them reported reduction. Bags under the eyes were reported by three people, two of whom reported reduction. This group was not asked about the flaccidity of the upper eyelid, because, due to safety reasons, the procedure cannot be performed on the eyelids. The use of protective glasses might have an impact on the lack of effectiveness in the case of shadows under eyes localized in the lower lid area. There are a number of studies showing the effectiveness of the IPL method in the treatment of symptoms associated with skin ageing. Apart from the remodelling of the dermis, IPL treatment also affects the reduction of discolorations [4, 27–32]. Local increase in the temperature during the treatment affects the blood flow in the treated area and improves metabolism. As a result, unnecessary metabolic products are removed, which may also indirectly influence the reduction of oedema or bags under the eyes [33].

In the conducted study, we confirmed the effectiveness of anti-ageing treatments. At the same time, showing that it seems necessary to expand the group with the effect of individual treatments against bags and dark circles under the eyes. Moreover, the obtained results confirm the utility of Multi Probe Adapter System – Cutometer in the realization of this research.

In the case of the non-ablation fractional laser treatment, bipolar radiofrequency and intensive pulse light, the side effects after procedures are minimal, mainly including transient erythema and oedema; other types of adverse reactions, such as blisters, are most often associated with an incorrect course of procedure [17, 19, 22, 34–36]. In this study, after non-ablative fractional laser treatment, some participants experienced redness, burning and oedema, which resolved after about 6 h. Additionally, most of them observed local, small scars which remained visible for a period of no more than 7 days. After the use of bipolar radiofrequency treatment, most participants demonstrated erythema, which resolved within 1 to 3 h after the procedure. After IPL treatment, some patients noticed redness, sometimes a feeling of burning up to 1 h after the procedure, as well as local hypersensitivity within the treatment area for about 3 to 12 h after the procedure. Some people with hyperpigmentation observed the intensification of pigmentation which lasted up to 7 days after therapy.

## Conclusions

Non-ablative fractional laser, bipolar radiofrequency and intense pulsed light are effective methods of rejuvenation of skin around the eyes. These types of treatments can be used to improve skin elasticity and reduce eye wrinkles. The most significant improvement of elasticity was demonstrated by laser therapy.

## References

[1] Radlanski RJ, Wesker KH (2015) The orbital region. In: Radlanski RJ, Wesker KH (eds) The face: pictorial atlas of clinical anatomy. Quintessence Publishing, Warszawa, pp 180–210

[2] Naouri M, Atlan M, Perrodeau E (2012). Skin tightening induced by fractional CO(2) laser treatment: quantified assessment of variations in mechanical properties of the skin. J Cosmet Dermatol.

[3] Kim EJ, Kwon HI, Yeo UC (2016). Lower face lifting and contouring with a novel internal real-time thermosensing monopolar radiofrequency. Lasers Med Sci.

[4] Shin JW, Lee DH, Choi SY (2011). Objective and non-invasive evaluation of photorejuvenation effect with intense pulsed light treatment in Asian skin. J Eur Acad Dermatol Venereol.

[5] Hedelund L, Due E, Bjerring P (2006). Skin rejuvenation using intense pulsed light: a randomized controlled split-face trial with blinded response evaluation. Arch Dermatol.

[6] Hantash BM, De Coninck E, Liu H (2008). Split-face comparison of the erbium micropeel with intense pulsed light. Dermatol Surg.

[7] Hedelund L, Bjerring P, Egekvist H (2006). Ablative versus non-ablative treatment of perioral rhytides. A randomized controlled trial with longterm blinded clinical evaluations and non-invasive measurements. Lasers Surg Med.

[8] Kapoor S, Saraf S (2010). Assessment of viscoelasticity and hydration effect of herbal moisturizers using bioengineering techniques. Pharmacogn Mag.

[9] Wickett RR (2001). Stretching the skin surface: skin elasticity. Cosmet Toiletries.

[10] Pierard GE, Nikkels-Tassoudji N, Pierard-Franchimont C (1995). Influence of the test area on the mechanical properties of the skin. Dermatology.

[11] Dobrev H (2000). Use of Cutometer to assess epidermal hydration. Skin Res Technol.

[12] Ahn S, Kim S, Lee H (2007). Correlation between a Cutometer and quantitative evaluation using Moire topography in age-related skin elasticity. Skin Res Technol.

[13] Ono I (2011). A study on the alterations in skin viscoelasticity before and after an intradermal administration of growth factor. J Cutan Aesthet Surg.

[14] Krueger N, Luebberding S, Oltmer M (2011). Age-related changes in skin mechanical properties: a quantitative evaluation of 120 female subjects. Skin Res Technol.

[15] Hwang YJ, Lee YN, Lee YW (2013). Treatment of acne scars and wrinkles in asian patients using carbon-dioxide fractional laser resurfacing: its effects on skin biophysical profiles. Ann Dermatol.

[16] Lee H, Yoon JS, Lee SY (2009). Fractional laser photothermolysis for treatment of facial wrinkles in Asians. Korean J Ophthalmol.

[17] Tretti Clementoni M, Lavagno R (2015). A novel 1565 nm non-ablative fractional device for stretch marks: a preliminary report. J Cosmet Laser Ther.

[18] Sukal SA, Chapas AM, Bernstein LJ (2008). Eyelid tightening and improved eyelid aperture through nonablative fractional resurfacing. Dermatol Surg.

[19] Bonan P, Campolmi P, Cannarozzo G (2012). Eyelid skin tightening: a novel ‘Niche’ for fractional CO_2_ rejuvenation. J Eur Acad Dermatol Venereol.

[20] Gold MH, Goldman MP, Rao J (2007). Treatment of wrinkles and elastosis using vacuum-assisted bipolar radiofrequency heating of the dermis. Dermatol Surg.

[21] Paasch U, Bodendorf MO, Grunewald S (2009). Skin rejuvenation by radiofrequency therapy: methods, effects and risks. J Dtsch Dermatol Ges.

[22] Fitzpatrick R, Geronemus R, Goldberg D (2003). Multicenter study of noninvasive radiofrequency for periorbital tissue tightening. Lasers Surg Med.

[23] Piccioni A, Fargnoli MC, Schoinas S (2011). Efficacy and tolerability of 5-aminolevulinic acid 0.5% liposomal spray and intense pulsed light in wrinkle reduction of photodamaged skin. J Dermatolog Treat.

[24] Moody MN, Landau JM, Goldberg LH (2012). Fractionated 1550-nm erbium-doped fiber laser for the treatment of periorbital hyperpigmentation. Dermatol Surg.

[25] Xu TH, Li YH, Chen JZ (2016). Treatment of infraorbital dark circles using 694-nm fractional Q-switched ruby laser. Lasers Med Sci.

[26] Atiyeh BS, Dibo SA (2009). Nonsurgical nonablative treatment of aging skin: radiofrequency technologies between aggressive marketing and evidence-based efficacy. Aesthetic Plast Surg.

[27] Kim JE, Kim BJ, Kang H (2012). A retrospective study of the efficacy of a new intense pulsed light for the treatment of photoaging: report of 70 cases. J Dermatolog Treat.

[28] Bitter PH (2000). Noninvasive rejuvenation of photodamaged skin using serial, full-face intense pulsed light treatments. Dermatol Surg.

[29] Mezzana P, Valeriani M (2007). Rejuvenation of the aging face using fractional photothermolysis and intense pulsed light: a new technique. Acta Chir Plast.

[30] Friedmann DP, Fabi SG, Goldman MP (2014). Combination of intense pulsed light, Sculptra, and Ultherapy for treatment of the aging face. J Cosmet Dermatol.

[31] Papageorgiou P, Clayton W, Norwood S (2008). Treatment of rosacea with intense pulsed light: significant improvement and long-lasting results. Br J Dermatol.

[32] Dierickx CC, Anderson RR (2005). Visible light treatment of photoaging. Dermatol Ther.

[33] Ash C, Town GA, Martin GR (2007). Preliminary trial to investigate temperature of the iPulse™ intense pulsed light (IPL) glass transmission block during treatment of Fitzpatrick II, IV, V, and VI skin types. Lasers Med Sci.

[34] Lee JW, Kim BJ, Kim MN (2010). Treatment of Periorbital Wrinkles Using a 2,790-nm Yttrium Scandium Gallium Garnet Laser. Dermatol Surg.

[35] Alam M, Dover JS (2003). Nonablative laser and light therapy: an approach to patient and device selection. Skin Therapy Lett.

[36] Stewart N, Lim AC, Lowe PM (2013). Lasers and laser-like devices: part one. Australas J Dermatol.

